# Robotic-assisted laparoscopic niche repair (RALNR): technique development and pregnancy-associated outcomes

**DOI:** 10.1007/s11701-025-02394-2

**Published:** 2025-05-29

**Authors:** Anne Muendane, Azadeh Babaei Bidhendi, Patrick Imesch, Isabell Witzel, Cornelia Betschart

**Affiliations:** 1 Department of Gynecology, University Hospital Zurich, University Zurich, Frauenklinikstrasse 10, 8091 Zurich, Switzerland; 2 Clinic for Gynecology, Bethanien Clinic, Zurich, Switzerland; 3 Department of Obstetrics and Gynecology, Medical University of Vienna, Vienna, Austria

**Keywords:** Cesarean section, Niche, Pregnancy rate, Reconstructive surgery, Robotic surgery, Ultrasound

## Abstract

Uterine scar defects after cesarean sections are increasingly common and elevate the risk of life-threatening complications in subsequent pregnancies. From various sonomorphological measurement parameters, the residual myometrial thickness (RMT) is crucial for predicting an obstetric complication in a subsequent pregnancy. A low RMT can be improved by surgical correction. The purpose of this paper is to present our technique for robotic-assisted laparoscopic niche repair (RALNR), to sonomorphologically characterize the niches pre- and postoperatively and to surveil subsequent symptoms and pregnancies. A cohort study of 35 patients with a niche and the wish to conceive, who had undergone RALNR between 05/2019 and 09/2023 at the university hospital of Zurich, was conducted. Sonomorphological parameters before and 6 weeks after surgery, as well as surgical, clinical and obstetrical outcomes were assessed. The mean widths and depths of the niche were significantly reduced (*p* < 0.001), width from 10.0 ± 3.5 mm preoperatively to 2.6 ± 3.4 mm postoperatively, and depths from 9.1 ± 3.7 mm preoperatively to 1.8 ± 2.6 mm postoperatively. RMT was significantly improved after RALNR (*p* < 0.001) with mean 1.5 ± 1.5 mm preoperatively compared to 8.3 ± 2.9 mm postoperatively. The pregnancy rate was 13 of 18 (77%), and 7 re-cesarean sections were performed at term. Following surgery, RMT is improved, and subsequent pregnancy rates are high. Larger prospective studies with different long-term obstetric outcomes are needed to determine the clinical significance of RALNR in subsequent pregnancies. This effort advances the field`s state of the art by demonstrating a successful technique for RALNR and its clinical feasibility in a symptomatic cohort.

## Introduction

The number of cesarean sections (CS) has increased steadily since the last four decades. It is estimated to comprise 30% of all deliveries by 2030, which means that 38 million cesareans will be performed every year [1]. This development has led to a concurrent rise in cesarean section defects (CSD), also known as niches or isthmoceles, as a common complication [2]. As highlighted in a recent review article on CSD, the reported prevalence of sonomorphologically detected niches exhibits significant variability, ranging from 24 to 70% [11]. Severe niches can result in cesarean scar pregnancy in early pregnancy, later in placenta spectrum disorders, and uterine rupture in the third trimester or during labor, all of which are potentially life-threatening conditions [3, 4]. Moreover, additional typical niche-related symptoms in non-pregnant women include abnormal uterine bleeding (AUB), pelvic pain, and secondary infertility with a negative impact on quality of life, influencing relationships, work capacity, sexuality, or self-esteem [5]. For these reasons, considerable attention has been devoted in recent years to the better understanding of niche development mechanisms, identifying risk factors and evaluating treatment options [6–10]. Low residual myometrial thickness (RMT) over the niche has been identified as the most reliable prognostic factor for uterine rupture or dehiscence in pregnant women [12, 13]. In terms of diagnosis, transvaginal ultrasound is recognized as an effective tool for evaluating niche size and RMT [4, 14, 15]. A standardized approach to niche assessment should be used as initially recommended by Naji et al. [2] and published in a Delphi procedure guideline in 2019 [16]. Niche evaluation is feasible starting 6 weeks after delivery [13, 17], providing similar results to an evaluation at 11–14 weeks of gestation [18]. Therefore, counseling and therapy of scar defects can be completed reliably before conception, where a surgical treatment of the niche is possible.

Various surgical techniques have been evaluated for repairing niches, including laparoscopy [19–21], hysteroscopy [20–23], combined laparoscopy, and hysteroscopy in the rendezvous technique [19, 21] or vaginal repair [19, 20]. These surgical methods are applied when non-invasive methods like medical treatment for bleeding disorders have failed in treating major niche-related symptoms, or when a pregnancy is desired, but RMT is reduced [24].

Robot-assisted laparoscopy is known to improve surgical results by enhancing fine motor skills through flexible instruments, providing greater variability in movements. Such advantages can be highly beneficial, especially in precisely excising defect zones and suturing delicate tissue like the myometrium. Robotic niche repair is an emerging field, however, and data on its immediate and pregnancy-related outcome is single-case based [25, 26].

The primary aim of this study is to describe our RALNR technique and assess its surgical success. Secondary objectives include reporting clinical outcomes such as symptom relief and pregnancy and delivery rates to contribute to the growing literature on niche repair.

## Methods

This observational study was conducted at the University Hospital Zurich and included 35 patients with severe niche after undergoing at least one cesarean section, who, desiring a future pregnancy, underwent RALNR between 05/2019 and 09/2023. During the period 3/2020 to 10/2020, no niche surgeries were performed due to COVID restrictions. The study protocol received its approval by the local ethics committee (BASEC-ID2022-02315), and general consent for analyzing clinical data was obtained from all participants of legal age. Patients with no general consent as well as patients with completed family planning were ineligible for this study.

Additionally, clinical and demographic data such as age at surgery, BMI, parity, number of CS, other previous surgeries, latency between CS and niche repair, indication for surgery, months of infertility prior to surgery, and endometriosis/adenomyosis as a concomitant disorder were gathered from the medical chart. If necessary, participants were contacted by telephone to assess the clinical and pregnancy outcomes.

### RALNR: three-layered myometrial closure technique in the anteflexed uterine position

RALNR was performed by two experienced surgeons (C.B. and P.I.) using a daVinci Xi surgical system (Intuitive Surgical Inc., USA). A single-shot prophylactic antibiosis with 2 g cefazolin iv was administered 20 min prior to surgery. After placing the patient in the lithotomy position, a dispersive electrode was attached on the right thigh. A Spackman manipulator (Hartmann AG, Switzerland) was inserted into the uterus. Laparoscopic approach through five trocars of 8–10 mm was used and the robotic arms of the daVinci Xi (Intuitive Surgical Inc., USA) were connected. The camera was installed at the umbilicus, a monopolar scissor and bipolar coagulation device were placed on the right and left side on the lower abdomen, and a smoke evacuation and a suction device placed more laterally.

**Fig. 1:**
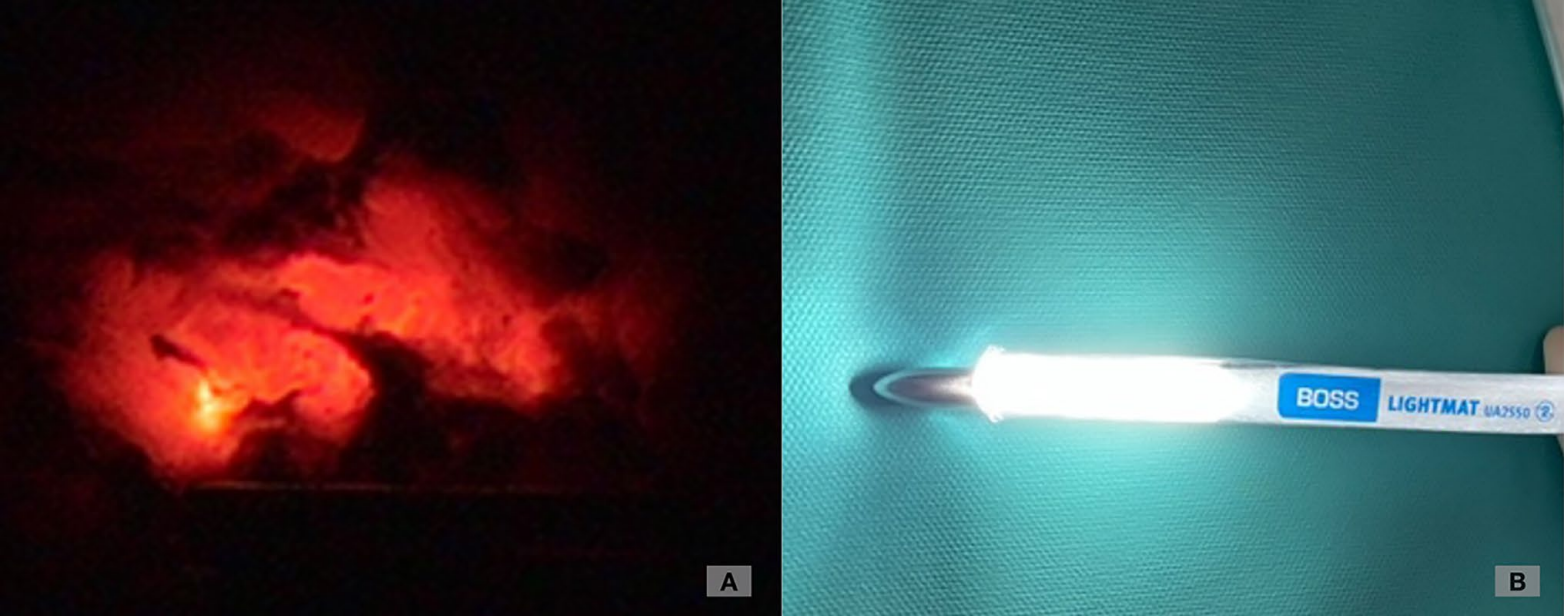
**Niche-identification with diaphanoscopy.** **A** Intraoperative image showing diaphanoscopy using a lighted Hegar dilator during robotic-assited laparoscopic repair of a uterine niche. The niche is visualized as the brightest area in the lower uterine segmanet, a phenomenon referred to as the ‘Halloween sign’. **B** Lighter Hegar dilator

**Fig. 2:**
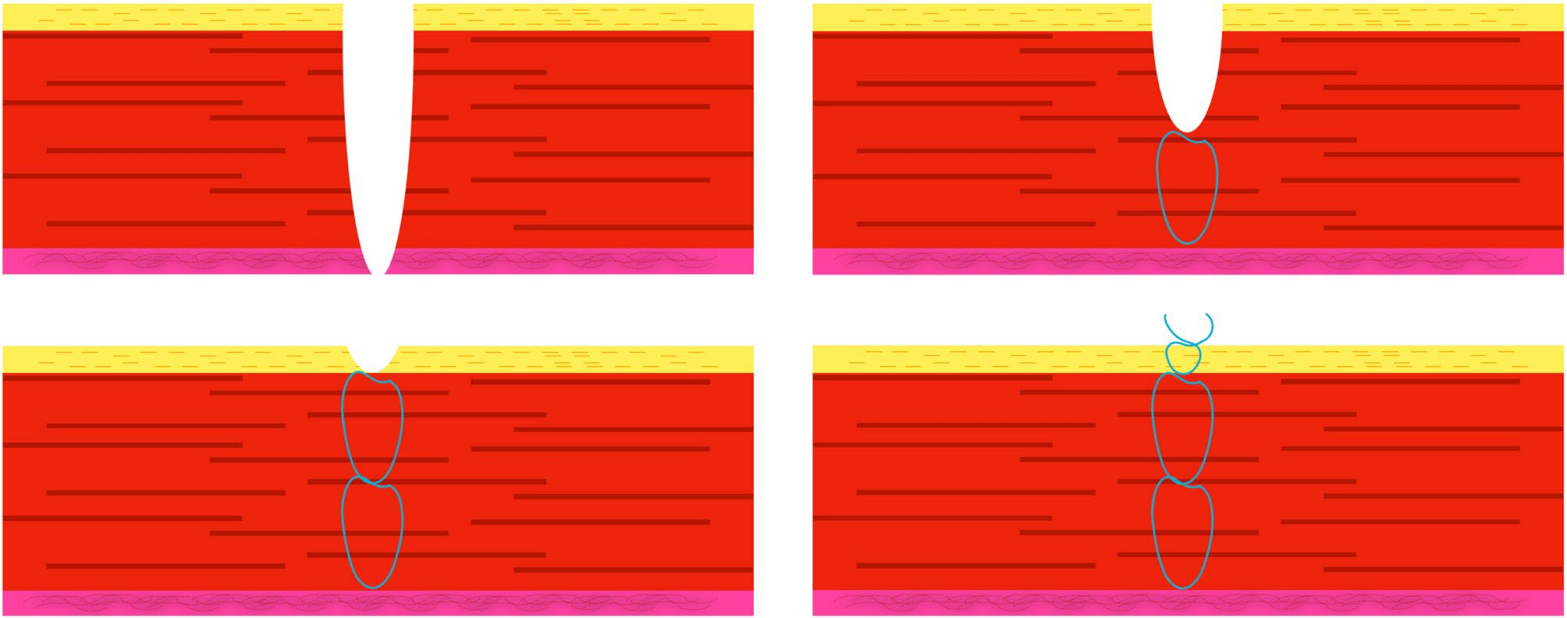
**Suture technique of RALNR.** First layer: V-lock 3-0 adapting first half of myometrium (red) without endometrium (pink). Second layer: V-lock 3-0 adapting second half of myometrium (red). Third layer: Vicryl 2-0 continous suture of serosa (yellow). *RALNR* robotic assisted laparoscopic niche repair

**Fig. 3:**
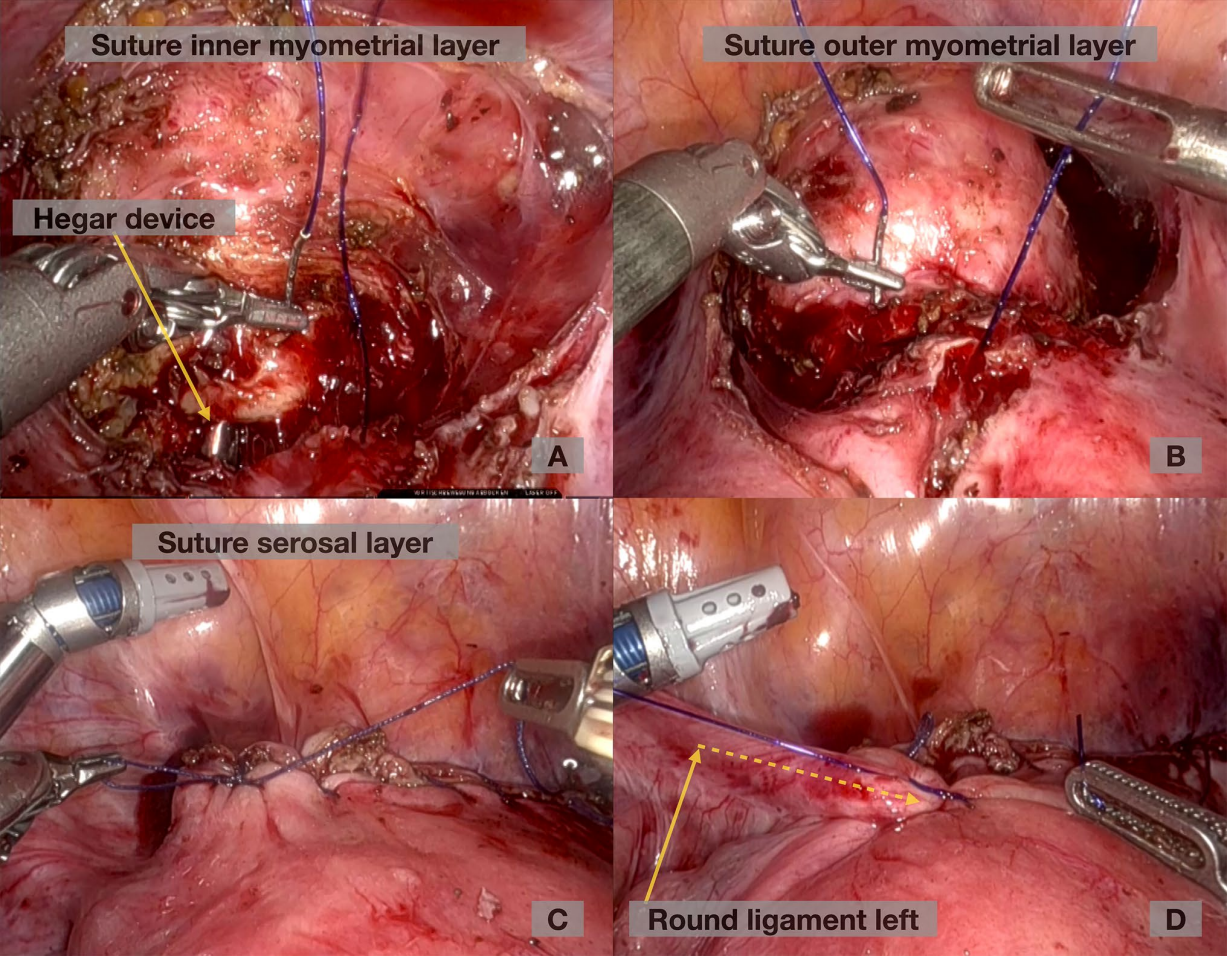
**Intraoperative presentsation of 3 layer suture technique of RALNR.** Intraoperative images demosntrating the three-layer suturing technique during RALNR. **A** First suture layer: closure of th inner myomeyrial layer, with guidance and visualizatin of the cervical channel by a Hegar. **B** Second suture layer:closure of the outer myometrial layer. **C** Third suture layer: cloaure of the serosal layer. **D** Round ligament temporarily sutured to the anterior uterine wall. *RALNR* robotic assisted laparoscopic niche repair

For the niche repair, the following surgical steps were applied:

1. Lancing of the bladder peritoneum underneath the round ligaments at the level of the scar tissue on both sides toward lateral.
2. Preparation of the posterior bladder wall distally and dissection of scar tissue if necessary to visualize the area of the niche.
3. Removal of the uterine manipulator and insertion of the lighted Hegar device LightMat (Ref Nr. UA 2550, LOT I05045) after dilation of the cervix up to Hegar 10.
4. Identification of the thinnest region via diaphanoscopy (see Fig. 1)
5. Spindle-shaped resection of the isthmocele under minimal coagulation to preserve the intact myometrium. The niche and a few millimeters of surrounding tissue were sent to histopathology.
6. Drainage of mucus was performed as well as excision of mucoceles, if present, to prevent pressure on the suture during wound healing.
7. Three-layer closure using running, barbed suture (V-Loc™ 3-0) for the two inner layers containing the first half of the myometrium, without endometrium, and a second outer myometrial layer, using Vicryl 2-0 continuous non-locked suture for the serosal layer (see Figs. 2 and 3).
8. Stitching the round ligaments to the anterior uterine wall using slow-resorbable polydioxanone sutures (PDS 2-0) to minimize tension on the scar and to obtain an anteflexed uterine position during wound healing for about 2–3 months.

Patients were discharged the first or second day after RALNR and were advised to use a reliable form of contraception for at least 6 months to ensure sufficient stabilization and healing of the scar tissue.

### Ultrasound assessment

Transvaginal ultrasound evaluation (Voluson S10 expert, GE Health Care, USA, RIC 5-9A-RS Realtime 4d Endocavity Probe, 4.0 MHz–10.0 MHz frequency) of the uterine cesarean scars at a preoperative visit and 6 weeks postoperatively were measured by one examiner (A.M.). As recommended by Naji et al. [2], widths, depths, residual myometrial thickness (RMT), distance to the internal cervical os as a surrogate marker for the location of uterotomy, as a lower incision site is associated with greater CSD [10], and uterine position were noted in a case file.

The identification of the internal cervical os by ultrasound was defined by Osser et al. [4].

### End points

The primary end point was the difference in RMT from pre- to postoperative, measured in millimeters and displayed in percentage (%). The secondary end points were change (difference) in widths and depths of the niche, also measured in millimeters and displayed in percentage (%). The distance niche to the inner cervical os was measured in millimeters and displayed as average in millimeters.

The secondary end point, pregnancy rate, is measured as occurrence of pregnancy (pregnancies in the individual participant) as yes or no and displayed as percentage (%). The secondary end points, symptom relief and delivery of a living child > 24 weeks of gestation, are measured binarily as present or not present and displayed as participants (*n* = *x*) with symptom relief/or live birth > 24 weeks of gestation in percentage (%).

### Statistical analysis

For statistical analysis, descriptive statistics for the characterization of the collective and Wilcoxon test for the dichotomous, dependent variables was applied using SPSS. A *p* value < 0.5 is considered significant.

To determine statistical robustness, two power analyses were performed with an alpha level of 0.05 in a two-tailed paired t-test for the primary end point of surgical success and for the secondary outpoint of adverse pregnancy outcomes with G*Power.

## Results

**Fig. 4:**
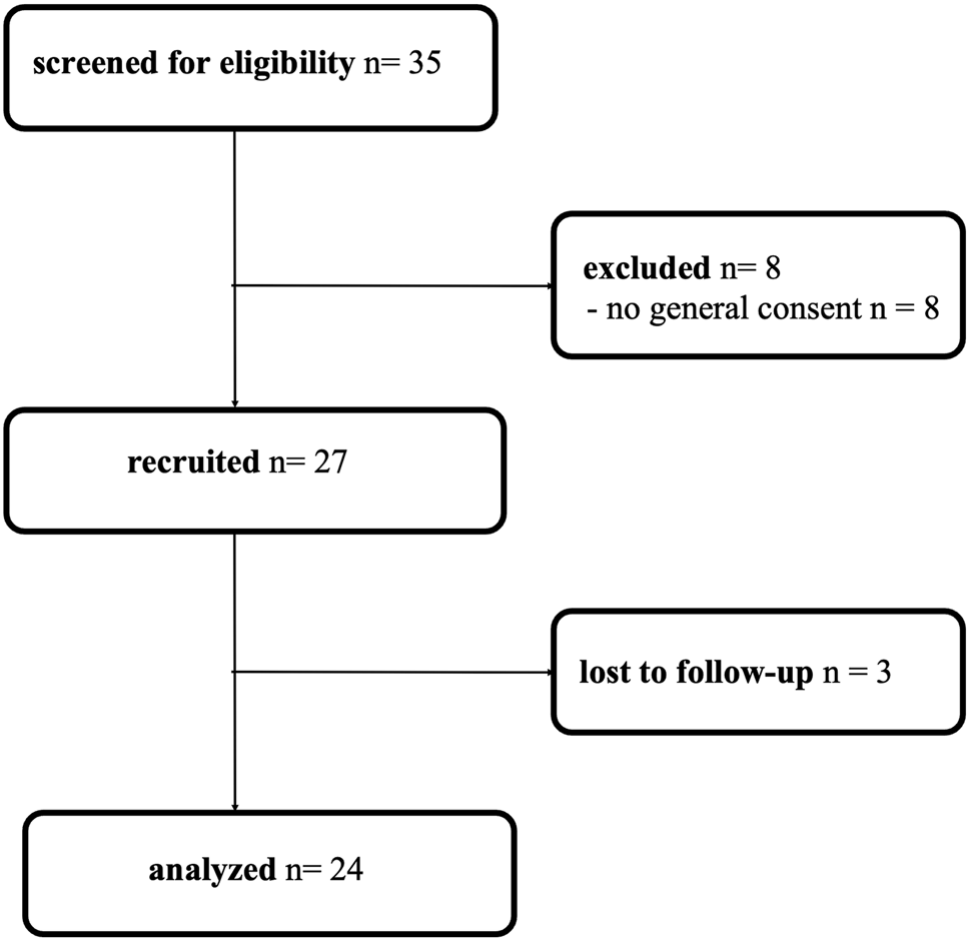
Flow chart

35 patients were screened for eligibility. Eight of these had to be excluded, as no general consent for further data use for research purposes was given, while three other women were lost to follow-up. Thus, 24 were eligible to be analyzed with full datasets (see Fig. 4).

### Demographic and obstetric data

The mean age was 34 years with a range of 26–43 years. BMI was 25 kg/m^2^ with a range of 18–35 kg/m^2^. The majority of participants were primiparous with a history of one CS; only six women had delivered twice by CS. Secondary indications for CS, such as stalled labor or abnormal fetal heart rate pattern, were more common than primary ones in 14/24 and 6/24 cases, respectively. In three cases, an emergency CS was performed at the last CS (Table 1).

**Table 1:** Demographic–obstetrical data and indication for RALNR

Number of participants, *n*	24
Age, years (mean, range)	34 (26–43)
BMI, kg/m^2^ (mean, range)	25 (18.9–35.6)
Parity	
• 1, *n*	17
• 2, *n*	6
• 3, *n*	1
Previous abdominal or uterine surgery	
• Laparoscopic niche repair	1
• Laparoscopic resection of endometriosis	2
• Diagnostic laparoscopy and chromopertubation	1
• Open appendectomy	2
• Hysteroscopic resection of uterine septum	1
• Curettage	2
Number of previous cesareans	
• 1, *n*	18
• 2, *n*	6
Indication for last cesarean section	
• Primary, *n*	7
• Secondary, *n*	14
• Emergency, *n*	3
Latency between CS and RALNR, months (mean, range)	36 (11–180)
Indication for RALNR	
• Wish for children alone, *n*	6
• Wish for children and secondary infertility, *n*	8
• Wish for children and symptoms, *n*	9
• Open family planning and pain alone, *n*	1
Duration of infertility until RALNR, months (mean, range)	16 (12–24)
Concomitant endometriosis or adenomyosis	9

The demographical–obstetrical data, previous abdominal surgeries, concomittant endometriosis, and indications for RALNR are shown

*RALNR* robotic-assisted laparoscopic niche repair, *BMI* body mass index, *n* number, *CS* cesarean section

Eight participants had a history of previous abdominal or uterine surgeries (Table 1). Most commonly, in 3/8 cases laparoscopic procedures for endometriosis or chromopertubation had been performed. One had already undergone a laparoscopic niche repair in the past. In two cases, a curettage, in two cases an open appendectomy, and one hysteroscopic resection of a uterine septum had been performed in the past.

Eight women had already tried to conceive before niche repair and experienced secondary infertility. Nine women reported abnormal uterine bleeding and/or dysmenorrhea or pain upon being asked. One woman opted for RALNR due to chronic pelvic pain and open family planning. The latency between (last) CS and RALNR was 36 months (11–180 months). The latency between the diagnosis of secondary infertility and surgical repair was much shorter, namely 16 months (12–24 months).

### Sonomorphological results and surgical characteristics

RMT as a primary end point had a mean of 1.5 ± 1.5 mm preoperatively and increased to 8.3 ± 2.9 mm postoperatively (*p* < 0.001),which represents a fivefold increase in thickness (553%), showing a significant improvement through RALNR (see Table 2).

**Table 2:** Sonomorphological results in transvaginal 2D ultrasound

	Preoperative (*n* = 24)	Postoperative (*n* = 24)	Difference (%)	*p*-value
Uterine position				
• Anteflexed	12	23	–	–
• Streched	2	0	–	–
• Retroflexed	10	1	–	–
Distance to cervical os, mm (mean)	3.9	3.9	0	–
RMT, mm (mean)	1.5	8.3	+553	< 0.001
Widths, mm (mean)	10.0	2.6	−74	< 0.001
Depths, mm (mean)	15.9	1.8	−89	< 0.001

Detailed information on pre- and postoperative changes in uterine position and sonomorphological parameters of the niche dimension is shown

*n* number, *mm* millimeters, *RMT* residual myometrial thickness

For a mean difference of 6.8 mm, and assuming a conservative estimate for the standard deviation of the paired differences as 3.27 mm, the resulting effect size (Cohen’s d for paired samples) is 2.08. With a sample size of 24, the statistical power exceeds 0.99. Therefore, the study is sufficiently powered to detect this difference with high confidence.

The preoperative mean niche width was 10.0 ± 3.5 mm and the postoperative 2.6 mm ± 3.4 mm (*p* < 0.001), which is a reduction of 74%. Mean depth was 15.9 ± 3.7 mm preoperatively and 1.8 ± 2.6 mm postoperatively (*p* < 0.001), i.e., a reduction of 89%. Niche widths and depths could both be significantly reduced through the intervention (see Table 2). A retroflexed uterine position was preoperatively present in 10/24 cases. Through RALNR, the position of nine of these uteri was changed to an anteflexed position at 6 weeks postoperatively.

The mean distance of the niche from the internal cervical os was 3.9 mm pre- and postoperatively.

Mean operating time was 145 min (range 103–244 min). Adhesiolysis of > 60 min was performed in two cases, whereas the preparation of the vesicouterine and paracervical adhesions or those containing the round ligaments occurred in 87% of cases (*n* = 21) and bowel or pelvic wall adhesion had to be removed in 37.5% (*n* = 9). Mucus was released from the niche in 79%. Concomitantly, in one case a myomectomy, in two cases a hysteroscopy (one diagnostic, one polypectomy), and in two cases a cystoscopy to preclude intraoperative bladder damage during extensive vesicouterine adhesiolysis were performed. Mean blood loss was 60 ml (range 20–400) (Table 3). In one patient, the blood loss was extended to 400 ml due to a fibroid of > 70 mm at the uterotomy site, which was intraoperatively removed. Neither transfusions nor conversion to laparotomy was needed. No surgical complications, such as bladder or bowel injury or postsurgical infections, occurred (see Table 3).

**Table 3:** Surgical characteristics

Number of participants, *n*	24
Operative time, min (mean, range)	145 (103–244)
Blood loss, ml (mean, range)	60 (20–400)
Conversion to laparotomy, *n*	0
Complications (transfusion needed, bowel or bladder injury, infections)	0
Concomittant surgery	
• Myomectomy	1
• Hysteroscopy	2
• Cystoscopy	2

Surgical characteristics including operation time, blood loss, and concomittant surgeries are shown

*n* number, *min* minutes

Histopathology of the niches revealed smooth muscle cells, fibrosis, endometrial cells, and endocervical glands, but no pregnancy retention tissue and no malignant or pre-malignant tissue.

### Clinical outcomes

Among the 23 women expressing a desire to conceive, 6 achieved the recommended 6-month interval between surgery and pregnancy only after the study end. 13/17 women became pregnant during the study period, which means a pregnancy rate of 77%. Of those, seven (54%) planned re-CS were performed. No preterm births or emergency CS occurred. Five women experienced early pregnancy loss (38%),  three of them lost more than one pregnancy in the first trimester (23%) (see Table 4). Two of the five participants had a history of recurrent pregnancy loss. No ectopic pregnancy occurred.

**Table 4:** Clinical outcomes on obstetrical and symptomatic parameters

	Number of eligible cases	Percentages (%)
Pregnancy rate	13/17	77
Live birth > 24 weeks of gestation	7/13	54
Pregnancy loss rate	5/13	38
Recurrent pregnancy loss rate	3/13	23
Relief of abnormal bleeding	5/6	83
Relief of pain/dysmenorrhea	2/4	50

Important end points such as clinical success rate, pregnancy rate and adverse pregnancy outcomes are shown

For the power analysis of early pregnancy loss, we evaluated the difference between the observed early pregnancy loss rate in our cohort (38%; 5/13 pregnancies) and a reference rate from the general population (15%). Using a one-sample proportion test, the calculated effect size (Cohen’s h) was 0.48. Due to the limited sample size (*n* = 13 pregnancies), the post hoc power was low (< 0.50), suggesting that our study is underpowered to detect statistically significant differences for this outcome.

In five of six women (83%) with bleeding disorders, symptom relief could be achieved by surgery. Two out of four patients with chronic pelvic pain reported satisfactory pain relief after surgery. Two others with dysmenorrhea experienced no relevant improvement through RALNR, but one of them had adenomyosis diagnosed intraoperatively.

## Discussion

### Main findings and interpretation

With this paper, we present a cohort study of patients who have undergone a surgical technique for RALNR. The key steps comprise the correct identification of the niche using diaphanoscopy, resection of the defective scar tissue, and a three-layer closure of the defect involving the subendometrial and myometrial layers, as well as serosa to achieve complete and stable closure of the wound. Furthermore, we assure the healing of the anterior uterine wall in an anteflexed position to prevent tension on the scar and recurrence of the niche.

Through RALNR, significant improvement of all sonomorphological end points, i.e., RMT as the most prognostically relevant measure [12] as well as improved widths and depths of the niche, was obtained. Pregnancy and delivery rates were high without complications other than early pregnancy loss, which most probably cannot be attributed to the niche repair, as spontaneous loss in the first trimester is estimated to present in 15–25% of all pregnancies [27], and moreover some of the affected women already had a history of recurrent pregnancy loss before RALNR. No CS pregnancy, uterine rupture, placenta accreta spectrum disorder, preterm birth, or emergency cesarean section occurred.

Additionally, satisfactory relief from a bleeding disturbance and chronic pelvic pain—but not dysmenorrhea—could be achieved in the majority of women affected. One out of two patients with dysmenorrhea was intraoperatively diagnosed with adenomyosis as a concomitant reason for her complaints. In a recent publication, endometriosis was found to be prevalent in 26.5% of women undergoing an isthmocele repair [28] and seems to play a role in CSD-related subfertility [29]. Otherwise, Timmermans et al. found in their study on laparoscopic niche repair that patients with adenomyosis and endometriosis should be carefully selected for niche repair, as symptom relief was not achieved in 40% of cases [30]. Our results are comparable to those published by Cardaillac et al. [31], with favorable impact of a RALNR on RMT, fertility rates, and gynecological symptoms. In their study, RMT improved from 1.6 mm to 4.3 mm within 3–6 months after surgery [31].

Our demographic data revealed a higher prevalence of secondary CS deliveries and a greater incidence of retroflexed uteri prior to surgical repair compared to afterward. Additionally, vesico-uterine adhesions were seen in 87% of patients. In half (12/24 cases) of our histological reports of the resected niche, endocervical glands were described, and in 79% of cases, mucus inside the niche was clinically present, indicating a cervical location of the cesarean incision. All of these factors have been hypothesized to be associated with impaired uterine wound healing and niche development in the past [4, 6, 8–10].

The extent and configuration of the niche, especially if endometrium is present within the niche, is associated with AUB [6, 14, 17, 32]. Vitale et al. (2020) showed that surgery improved abnormal bleeding in 80% of patients, consistent with our 83% success rate. [20] He et al. concluded in a systematic review and meta-analysis including four RCTs and six observational studies that combined laparoscopic and hysteroscopic approaches reduce bleeding and niche size more effectively, likely due to diaphanoscopy-aided precision [19].

If RMT is insufficient, laparoscopy is one of the preferred approaches to yield a satisfactory and functional anterior uterine wall thickness [24]. A robust multi-layer closure, in particular, seems to be of great importance in achieving a postoperatively sufficient RMT. The 3D vision of the robotics device supports tissue layer recognition and seems advantageous compared with classic laparoscopy with its stereotypical motion sequences.

Overall, available data on anatomical findings and outcomes of robot-assisted laparoscopic niche repair (RALNR) are very limited. In particular, only four published studies on RALNR have been published so far, of which three are case reports introducing a robot-assisted niche repair technique [33–35]. One is a retrospective cohort study, which showed a promising improvement in RMT, fertility rates, and gynecological symptoms [31]. Additionally, two publications address the concomitant excision of a cesarean scar pregnancy and niche correction with the robot, of which one is a case report with subsequent spontaneous conception after 18 months and uneventful pregnancy resulting in a scheduled re-cesarean section [25]. The other is a case series of 14 patients with a postoperative pregnancy rate of 64% with 8/9 planned term ee-cesareans and one recurrent cesarean scar pregnancy resulting in hysterectomy at 33 weeks of gestation [36].

### Impact on daily clinical practice and further research

RALNR might become a suitable surgical therapeutic option for women with a uterine niche and a wish for further children. For quality control and as a reliable imaging parameter, we recommend performing transvaginal ultrasound assessment of the uterine scar routinely at 6 weeks after CS or at the next regular gynecological checkup to identify women with a severe niche. In cases of an RMT < 3 mm defined as a severe scar defect, the estimated uterine rupture or dehiscence rate is OR 26.05 (2.36–287.61) compared with a small or no scar defect [12]. Reducing this risk is what RALNR is aimed at. Our study gives physicians the opportunity to discuss treatment options early and perform surgery before a subsequent conception. It is not powered to detect uterine scar rupture rates after RALNR. Whether RALNR prevents uterine ruptures cannot be answered with our collective. In addition, biomechanical properties of scar tissue such as stiffness may play an important role in adverse events like uterine rupture in a subsequent pregnancy [37], which could become future research questions. Currently, there is little data correlating imaging and clinical outcome in a subsequent pregnancy [12, 13] or after RALNR [31].

### Strengths and limitations

The main strengths of this study are that all RALNRs were performed by two experienced surgeons, and that a systematic, second look ultrasound assessment was made by one examiner. Further strengths are that not only sonomorphologic features, but also clinical outcomes, such as symptom relief, pregnancy, and delivery rates, were assessed.

Obvious limitations are the small number of participants and the single-center experience. We acknowledge that our current sample of 13 women with one or more early pregnancy losses is underpowered to detect statistically significant differences for this secondary outcome. Although the observed loss rate (38%) appears elevated compared to the general population rate (15%), post hoc power analysis indicated that at least 39 pregnancies would be required to reliably detect a statistically significant difference between pregnancy loss following RALNR and that in the general population. Prospective, long-term observation will be necessary to answer the real-life perspective of pregnancy rates as well as obstetrical consequences for placenta spectrum disorders, ectopic pregnancies, and uterine ruptures in subsequent pregnancies after RALNR.

## Conclusion

Robotic-assisted laparoscopic niche repair (RALNR) improves RMT and bleeding disorders significantly. In addition, high postsurgical pregnancy rates could be observed. It is imperative, however, that further studies in larger, multi-center collectives with long-term follow-up be conducted to explore the clinical relevance of RALNR on following pregnancies.

## Data Availability

No datasets were generated or analysed during the current study.
